# Regulation of gap junction conductance by calcineurin through Cx43 phosphorylation: implications for action potential conduction

**DOI:** 10.1007/s00424-016-1885-7

**Published:** 2016-10-19

**Authors:** Rita I Jabr, Fiona S Hatch, Samantha C Salvage, Alejandro Orlowski, Paul D Lampe, Christopher H Fry

**Affiliations:** 1 School of Biosciences and Medicine, Faculty of Health and Medical Sciences, University of Surrey, Guildford, GU2 7XH UK; 2 Institute of Cardiovascular Research, Ashford & St Peter’s NHS Foundation Trust, Surrey, Chertsey, KT16 0PZ UK; 3 Fred Hutchinson Cancer Research Center, 1100 Fairview Avenue North, Seattle, WA 98109 USA; 4 School of Physiology, Pharmacology & Neuroscience, University of Bristol, BS8 1TD Bristol, UK

**Keywords:** Connexin 43, Gap junction conductance, Calcineurin, Conduction velocity

## Abstract

Cardiac arrhythmias are associated with raised intracellular [Ca^2+^] and slowed action potential conduction caused by reduced gap junction (GJ) electrical conductance (*G*j). Ventricular GJs are composed of connexin proteins (Cx43), with *G*j determined by Cx43 phosphorylation status. Connexin phosphorylation is an interplay between protein kinases and phosphatases but the precise pathways are unknown. We aimed to identify key Ca^2+^-dependent phosphorylation sites on Cx43 that regulate cardiac gap junction conductance and action potential conduction velocity. We investigated the role of the Ca^2+^-dependent phosphatase, calcineurin. Intracellular [Ca^2+^] was raised in guinea-pig myocardium by a low-Na solution or increased stimulation. Conduction velocity and *G*j were measured in multicellular strips. Phosphorylation of Cx43 serine residues (S365 and S368) and of the intermediary regulator I1 at threonine35 was measured by Western blot. Measurements were made in the presence and absence of inhibitors to calcineurin, I1 or protein phosphatase-1 and phosphatase-2.

Raised [Ca^2^^+^]_i_ decreased *G*j, reduced Cx43 phosphorylation at S365 and increased it at S368; these changes were reversed by calcineurin inhibitors. Cx43-S368 phosphorylation was reversed by the protein kinase C inhibitor chelerythrine. Raised [Ca^2+^]_i_ also decreased I1 phosphorylation, also prevented by calcineurin inhibitors, to increase activity of the Ca^2+^-independent phosphatase, PPI. The PP1 inhibitor, tautomycin, prevented Cx43-365 dephosphorylation, Cx43-S368 phosphorylation and *G*j reduction in raised [Ca^2+^]_i_. PP2A had no role. Conduction velocity was reduced by raised [Ca^2+^]_i_ and reversed by calcineurin inhibitors. Reduced action potential conduction and *G*j in raised [Ca^2+^] are regulated by calcineurin-dependent Cx43-S365 phosphorylation, leading to Cx43-S368 dephosphorylation. The calcineurin action is indirect, via I1 dephosphorylation and subsequent activation of PP1.

**Electronic supplementary material:**

The online version of this article (doi:10.1007/s00424-016-1885-7) contains supplementary material, which is available to authorized users.

## Introduction

Propagation of the cardiac action potential (AP) between adjacent ventricular myocytes depends on gap junctions (GJs), located at intercalated discs and composed of connexin phosphoproteins, (Cx), mainly the isoform Cx43. Reduced AP conduction velocity, which potentially leads to re-entrant arrhythmias, is associated with a decrease of GJ unitary electrical conductance (*G*_j_) [7]. Several factors modulate *G*_j_ including an increase of the intracellular Ca^2+^ concentration ([Ca^2+^]_i_) or altered Cx43 phosphorylation [4, 14, 25, 31]. Myocardial hypoxia or ischaemia and conditions such as hypertrophy are associated with reduced AP conduction, raised intracellular [Ca^2+^] and GJ uncoupling [2, 26, 32, 34, 38]. However, the intracellular pathways for GJ uncoupling are unclear, although a change to the phosphorylation status of Cx43 has been implicated [20, 30, 35].

Alteration to Cx43 phosphorylation status results from changes to the activities of protein kinases (PKs) and/or phosphatases (PPs). Cx43 is targeted by several serine-threonine PKs, such as PKC, PKA and PKG, that either increase or decrease *G*_j_ [6, 25]. Cx43 protein is rich in serine (S) residues and phosphorylation of several S residues modulates *G*_j_, for example, at S306, S365 and S368 [27]. In particular, under physiological conditions S365 is predominant in its phosphorylated form (pS365), and this has been proposed to mask and prevent phosphorylation of S368 [39]. During myocardial ischaemia, pS365 is dephosphorylated to allow phosphorylation of S368 by PKCε [9, 10] and this is associated with a reduced *G*_*j*_ [33]. However, the identity of the PPs which dephosphorylate S365 is unknown. There are several candidates, for example Ca^2+^-independent serine-threonine PPs, such as PP1 and PP2A, modulate AP conduction in cardiac pathologies such as heart failure and acute ischaemia [1, 22]. However, the contribution of the Ca^2+^-calmodulin-dependent serine-threonine PP calcineurin (Cn) is unknown, although its activity increases in pathologies associated with arrhythmias [46].

Cn regulates the activity of many intracellular enzymes, including PKC and PP1 [5, 11]. Cn has also been implicated in the pathogenesis of cardiac arrhythmias associated with pathologies such as hypertrophic cardiomyopathy and aortic stenosis [28, 37]. However, the relationships between raised [Ca^2+^]_i_, Cn action, Cx43 phosphorylation state, *G*_j_ and AP conduction have not been characterised in myocardium. We hypothesised that with acute elevation of [Ca^2+^]_i_ Cn, in synergy with PKC, controls Cx43 phosphorylation to decrease *G*_j_ and slow AP conduction, with possible intermediate roles for PP1 and PP2A. Intracellular [Ca^2+^] was elevated in isolated ventricular myocardial preparations by reducing the extracellular [Na^+^] and by increasing stimulation rate. Calcineurin activation by Ca^2+^ is sufficiently rapid and sensitive that both interventions are sufficient to activate this protein phosphatase [36, 42].

## Materials and methods

### Isolated preparations and solutions

Dunkin-Hartley guinea pigs (400–500 g) were killed by cervical dislocation and the heart was rapidly excised in accordance with UK Guidelines in *The Operation of Animals (Scientific Procedures) Act*, 1986. Left ventricular (LV) papillary muscles or trabeculae (0.5–0.9 mm diameter, 5–7 mm long) were dissected immediately for experiments.

Control Tyrode’s solution contained mM NaCl 118, KCl 4.0, NaHCO_3_ 24, MgCl_2_ 1.0, CaCl_2_ 1.8, NaH_2_PO_4_ 0.4, glucose 6.1, and Na pyruvate 5.0, gassed with 95%O_2_/5%CO_2_, pH 7.40 ± 0.03. Low-Na Tyrode’s (29.4 mM Na) was similar except that NaCl was replaced by TrisCl (pH to 7.4 with 1 M HCl). Two Cn inhibitors were used: (i) cyclosporin-A (CysA; Calbiochem, UK), diluted from a 10 mM DMSO stock solution to a final concentration of 5 μM and (ii) the highly selective, cell-permeable Cn autoinhibitory peptide (CAIP; Calbiochem, UK), a peptide with similar amino acid sequence to the Cn autoinhibitory domain. A final concentration of 50 μM was freshly prepared from an aqueous 100 mM stock solution. The PKC inhibitor, chelerythrine (2 μM), was prepared from a DMSO stock solution (20 mM). PP1 inhibitor, tautomycin (5 nM), was prepared from a PBS stock solution (65.2 μM). PP2A inhibitor, fostriecin (100 nM), was prepared from a PBS stock solution (22.1 μM). All chemicals were from Sigma-Aldrich (UK) unless otherwise stated.

### Experimental protocols

Two interventions were used to increase [Ca^2+^]_i_,:(i) superfusion with low-Na Tyrode’s solution to elevate [Ca^2+^]_i_ via the Na^+^/Ca^2+^ exchanger; ii) an increase of electrical stimulation frequency from 1 up to 5 Hz. The effects of Cn, PKC or PP1 inhibitors on GJ conductance (Gj) and Cx43 phosphorylation status were measured in control conditions and during raised [Ca^2+^]_i_.

### Measurement of longitudinal impedance

*G*_j_ was measured with preparations in an oil-gap chamber and calculated from the frequency-dependent (0.02–300 kHz) total longitudinal impedance, *z*_i_—the method and its validation have been detailed elsewhere [3, 8]. After control readings in Tyrode’s solution, preparations were exposed to low-Na solution to raise the intracellular [Ca^2+^], with or without Cn or PP1/PP2A inhibitors, for 20–30 min before new readings were taken. Tyrode’s solution was then reapplied for final control measurements.

### Western blots

Western blot analysis was performed, as previously described with slight modification [40]. Hearts were perfused, using a Langendorff technique, for 10 min with Tyrode’s or low-Na Tyrode’s solutions in the absence or presence of Cn, PKC, PP1 or PP2A inhibitors. The LV was then rapidly cut off and snap frozen in liquid N_2_. Whole tissue protein lysate (30 μg) from each sample was prepared and then resolved by 12 % polyacrylamide SDS-PAGE and transferred to polyvinylidene difluoride membranes (PVDF; Invitrogen, UK). Membranes were blocked with an Odyssey blocking buffer (LI-COR Biosciences, Ltd., UK), probed with primary antibody (1:1000 dilution), then washed and incubated with secondary antibodies (1:10,000 dilution). Membranes were then stripped with a stripping buffer, washed and probed with another primary antibody followed by a secondary antibody. Resolved protein bands were imaged using an Odyssey infrared imaging system (UK) and then quantified with the Image-J software (NIH, version 1.4 K) in arbitrary units. The quantified band densities of pS368-Cx43 and pS365-Cx43 were normalised to corresponding total Cx43 bands. Similarly, the band densities of phosphorylated PP1 inhibitor-1 at threonine 35 (pThr35-I1) were normalised to total inhibitor-1. Total protein bands were normalised to corresponding glyceraldehyde 3-phosphate dehydrogenase (GAPDH) band density (used as a loading control). Faint GAPDH bands were apparent in figures illustrating stripped membranes. Each sample is shown in triplicate in the relevant figures.

### Measurement of AP morphology and conduction velocity

Preparations were secured at one end to a fixed hook and the other to an isometric force transducer in a horizontal tissue bath and superfused at 4 mL/min with Tyrode’s solution. Preparations were electrically stimulated with 50–100 μs pulses via Ag/AgCl bipolar electrodes on one end of the preparation [18]. Stimulating conditions were 1, 2 or 5 Hz in Tyrode’s solution and 1 Hz in low-Na solution. Conducted APs were recorded with multiple, separate downstream impalements using 3 M KCl-filled microelectrodes at known distances, *d*, from the stimulating electrodes. Conduction velocity (CV) was calculated from the difference in latency (∆*t*) recorded by two separate microelectrode impalements, distance ∆*d* apart, as the ratio ∆*d*/∆*t*. At least five separate measurement pairs were made per preparation. To elicit APs in low-Na solution, stimulus duration (0.5–1.0 ms) was increased. In all preparations, CV was measured with a stimulus voltage 1.5 times the threshold value. Values of the maximum rate of depolarisation during the AP upstroke (d*V*/d*t*_max_) and the time constant of the AP subthreshold region (AF foot, *τ*_ap_, [8]) were also recorded.

### Measurement of the intracellular [Ca^2+^] ([Ca^2+^]_i_)

The change of [Ca^2+^]_i_ with low-Na solution was measured in trabeculae with Ca^2+^-selective microelectrodes, filled with the Ca^2+^-ionophore ETH 1001 (Fluka Chemicals, UK) and in conjunction with 3 M-KCl-filled microelectrodes to record separately the membrane potential. Methods of manufacture, recording and calibration have been reported previously [16]. Dynamic changes to [Ca^2+^]_i_ with pacing were measured in freshly dispersed ventricular myocytes prepared by collagenase enzymatic dispersion using a Langendorff technique. Ventricular myocytes were loaded with Fura-2 (5 μM), superfused at 36 °C with Tyrode’s solution in a chamber on the stage of an inverted microscope. Cells were illuminated from a xenon-arc lamp that produces a continuous and uniform spectrum across the visible region. Excitation of the fluorochrome alternately at 340 and 380 nm was provided by interposing spectral band-pass filters (340 ± 5 and 380 ± 5 nm) within the light path, mounted in a wheel spinning at 32 Hz. Fluorescent light was recorded between 410 and 510 nm with a photomultiplier tube and output sample-and-hold amplifiers coordinated to the frequency of the spinning wheel. The ratio of emission intensity when illuminated at the two frequencies (*R*_340/380_) was used as an index of the intracellular [Ca^2+^] [41].

### Statistical analyses and calculations

Electrophysiological data are mean ± SEM (*n* preparations), as several measurements of all variables were made in each preparation. For Western blots, data are mean ± SD as one observation per preparation was made. Group comparisons used two-way ANOVA, with post hoc Bonferroni’s tests. The null hypothesis was rejected at *p* < 0.05: * vs control (Tyrode’s solution), # vs low-Na solution; **p < 0.01, ***p < 0.001. Cable calculations (see “Discussion” section) used Eq. 1 [7].

1 $$ {G}_{\mathrm{i}}=\left(2.{\tau}_{ap}.{C}_{\mathrm{m}}.C{V}^2\right)/a $$

where *G*_i_ is the total intracellular conductance, *τ*_ap_ is the time constant of the subthreshold base of the AP, *C*_m_ is the specific membrane capacitance (1 μF/cm^2^) and *a* is the cell radius (10.5 μm). Gap junction conductance, *G*_j_, was calculated from 1/*G*_j_ = 1/*G*_i_ − 1/*G*_c_, where *G*_c_ is the cytoplasmic conductance (5.9 mS/cm*—*see “Results” section).

## Results

### *G*_j_ with raised intracellular [Ca^2+^], [Ca^2+^]_i_—action of calcineurin inhibitors

**Fig. 1:**
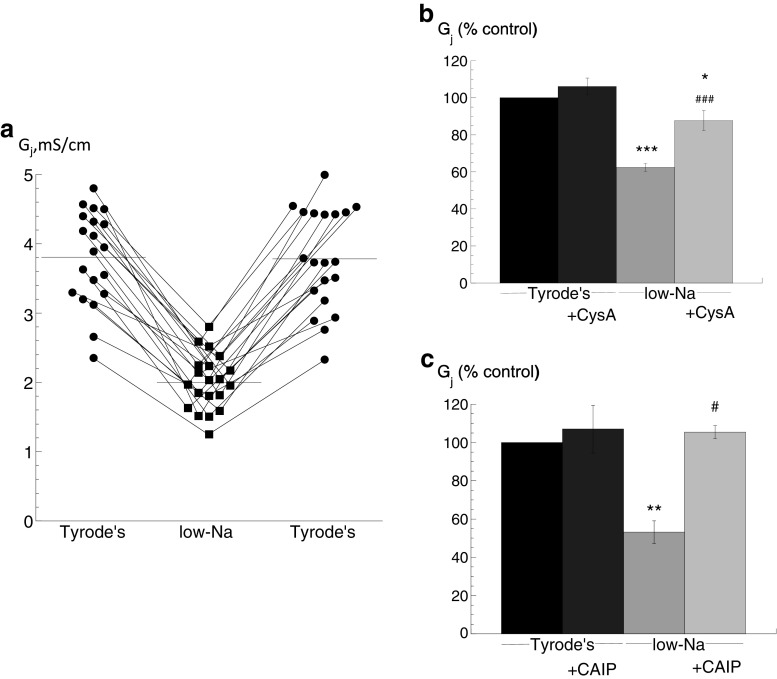
Low-Na solution on gap junction conductance (G_j_): effect of calcineurin inhibitors. **a** Values of *G*_j_ in Tyrode’s (control) before and after exposure to low-Na solution, data from 20 separate preparations. **b** Effect of cyclosporin-A (CysA, 5 μM) in Tyrode’s or low-Na solution on *G*_j_, data expressed as a percentage of the value in Tyrode’s solution (control), *n* = 5. **c** Effect of calcineurin-inhibitory peptide (CAIP, 50 μM) in Tyrode’s or low-Na solution on *G*_j_, *n* = 5 *p < 0.05 vs Tyrode’s; **p < 0.01 vs Tyrode’s; ***p < 0.001 vs Tyrode’s; #*p* < 0.05 vs low-Na; ###*p* < 0.0001 vs low-Na

The central hypothesis under test is that a raised [Ca^2+^]_i_ decreases *G*_j_ via a Cn-dependent pathway. Direct measurement of *G*_j_ in a multicellular preparation showed a reversible reduction when [Ca^2+^]_i_ was raised by superfusion with a low-Na solution (Fig. 1a). The attached supplement shows that the [Ca^2+^]_i_ as measured in similar preparations with ion-selective microelectrodes was significantly increased from 85 ± 10 to 405 ± 105 nM (*n* = 4) in the low-Na solution. This rise was sustained in these preparations for the period used to measure changes to *G*_j_ and conduction velocity, i.e. 20–30 min. In low-Na solution, the mean value of *G*_j_ was reversibly reduced from 3.80 ± 0.16 to 2.00 ± 0.09 mS/cm, *n* = 20, *p* < 0.0001, a change to 53.5 ± 2.5 % of that in Tyrode’s solution. Cytoplasmic conductance, *G*_c_, was unaffected by low-Na solution (5.94 ± 0.71 vs 5.65 ± 1.01 mS/cm, *n* = 20) or by any other intervention. The reduction of *G*_j_ in low-Na solution was partly prevented by cyclosporin-A (CysA; *n* = 5; Fig. 1b) or completely prevented by the more selective Cn inhibitor CAIP (*n* = 5; Fig. 1c); CysA and CAIP had no significant effect on *G*_j_ in Tyrode’s solution.

### Low-Na solution and S368-Cx43 phosphorylation (pS368)—action of Cn or PKC inhibitors

**Fig. 2:**
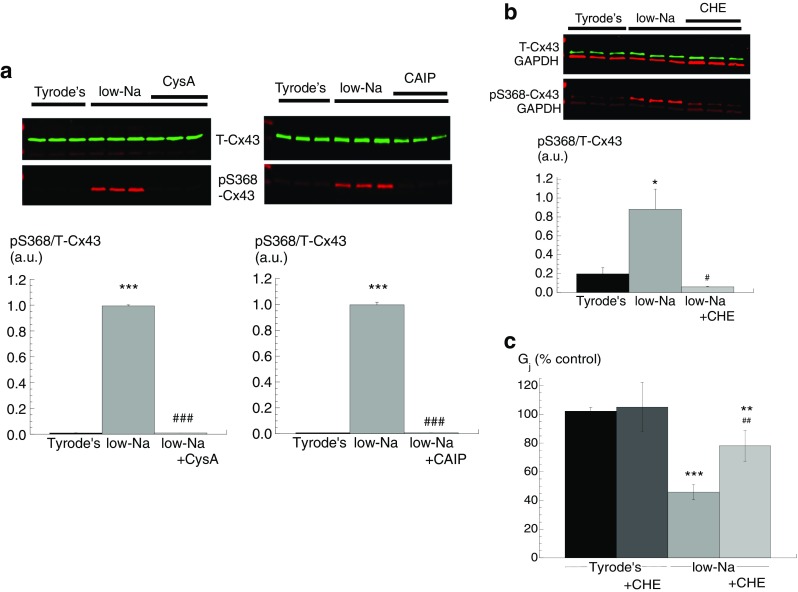
S368-Cx43 phosphorylation and gap junction conductance, G_j_, in low-Na solution: effect of calcineurin and PKC inhibitors. **a** Western blots of phosphorylated pS368-Cx43 (pS368-Cx43) in control and low-Na solution, effect of CysA (*left panels*) and CAIP (*right panels*). Band densities normalised to total Cx43 (T-Cx43) levels in the *lower panel*. **b** Western blots of phosphorylated S368-Cx43 (pS368-Cx43) in control Tyrode’s and low-Na solution, effect of chelerythrin (CHE, 2 μM). Band densities normalised to total Cx43 (T-Cx43) levels in the *lower panel*. GAPDH levels are also shown as a housekeeping protein. **c** Effect of chelerythrine (CHE, 2 μM) in Tyrode’s or low-Na solution on *G*_j_ and normalised to levels in Tyrode’s solution. *p < 0.05 vs Tyrode’s; **p < 0.01 vs Tyrode’s; ***p < 0.001 vs Tyrode’s; #*p* < 0.05 vs low-Na solution; ##*p* < 0.01 vs low-Na; ###*p* < 0.001 vs low-Na (*n = 4*)

The possible role of Cx43 phosphorylation, in particular at pS368, was examined as an underlying mechanism mediating decreased *G*_j_ in low-Na solution. A significant increase of pS368-Cx43, normalised to total Cx43 (T-Cx43), was measured in low-Na solution when compared to Tyrode’s. The increase was reversed, partially with CysA (*n* = 5) and completely with CAIP (*n* = 3, Fig. 2a), suggesting a role for Cn in this rise. T-Cx43 protein quantity was similar in all conditions, and when themselves, they were normalised to GAPDH levels were similar in all interventions.

The actions of the PKC inhibitor, chelerythrine (CHE, 2 μM), on pS368-Cx43 protein expression levels were measured in low-Na Tyrode’s. CHE reversed the increase of pS368-Cx43 expression induced by low-Na solution to values not significantly different from control Tyrode’s solution (*n* = 5, Fig. 2b). In addition, the action of CHE on *G*_j_ was tested. CHE had no effect on *G*_j_ in control Tyrode’s solution. However, the significant reduction of *G*_j_ by low-Na solution was also reversed by CHE (*n* = 5, Fig. 2c). These data show that in low-Na solution, there is increased Cx43 phosphorylation at S368, associated with a decrease of *G*_j_. A cooperative role for PKC and Cn is suggested, whereby a Cn-dependent pathway enables PKC to phosphorylate Cx43 at S368 and reduce *G*_j_.

### Cn inhibitors and S365-Cx43 (pS365-Cx43) phosphorylation in low-Na solution

**Fig. 3:**
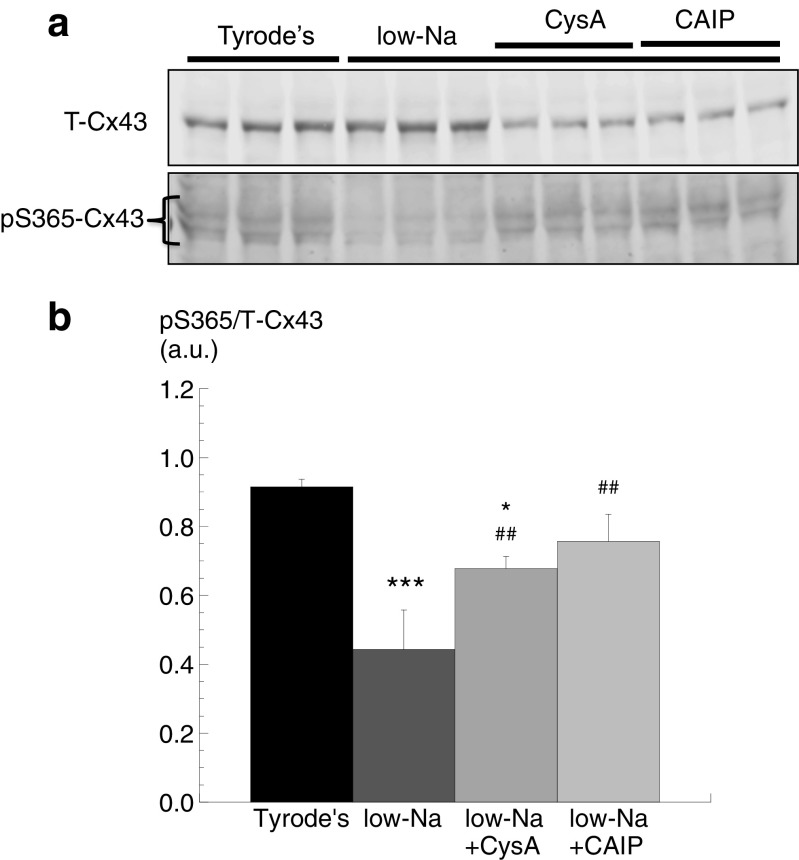
S365-Cx43 phosphorylation in low-Na solution: effect of Cn inhibitors. **a** Western blots of T-Cx43 and pS365-Cx43 in control and in low-Na solution in the absence or presence of CysA or CAIP. **b** Band densities of pS365-Cx43 normalised to T-Cx43 in the different conditions. *p < 0.05 vs Tyrode’s; ***p < 0.001 vs Tyrode’s; ##*p* < 0.01 vs low-Na (*n = 3*)

Phosphorylated S365-Cx43 has been proposed as a gatekeeper site that regulates S368 phosphorylation [39]. Therefore, it was tested if Cn had a role in the dephosphorylation of pS365-Cx43. The level of pS365-Cx43 was significantly lower in low-Na solution compared with Tyrode’s (*n* = 3; Fig. 3a, b). Moreover, CysA or CAIP, when added to the low-Na solution, reversed this decline, completely with CAIP and partially with CysA. These data are consistent with a Cn-dependent pathway dephosphorylating pS365-Cx43 when [Ca^2+^]_i_ is raised, which allows PKC to then phosphorylate S368 and hence reduce *G*_j_.

### A direct or indirect action of Cn activation on Cx43 phosphorylation status

The data thus far do not distinguish between a direct or indirect effect of Cn on pS365-Cx43 dephosphorylation. Ca^2+^-independent PP1 is bound in an inactive state to phosphorylated I1 (pThr35-I1). One target for Cn is pThr35-I1, which when dephosphorylated will release activated PPI [11]. This potential pathway was examined by measuring the effect of Cn inhibitors on pThr35-I1 levels when [Ca^2+^]_i_ was raised.

**Fig. 4:**
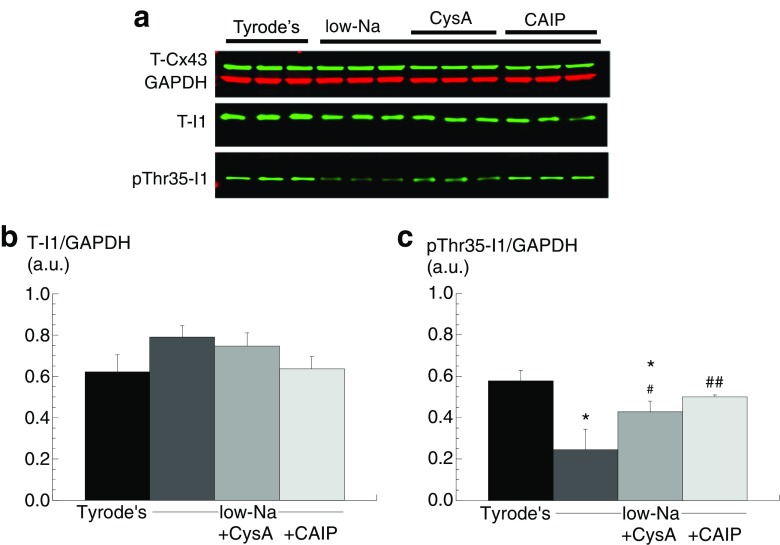
Thr35-I1 phosphorylation in low-Na solution: effect of Cn inhibitors. **a** Western blots of T-Cx43, GAPDH, T-I1 and pThr35-I1 in control and in low-Na solution in the absence or presence of CysA or CAIP. **b** Band densities of T-I1 normalised to GAPDH. **c** Band densities of pThr35-I1 normalised to T-I1 in different conditions. *p < 0.05 vs Tyrode’s; #*p* < 0.01 vs low-Na ; ##*p* < 0.01 vs low-Na (*n = 3*)

Total I1 protein, normalised to GAPDH, was similar in control and low-Na solutions and also in the presence of CysA or CAIP (*n* = 3, Fig. 4a, b); T-Cx43 was also constant throughout. In low-Na solution, pThr35-I1 levels were significantly reduced. This reduction was partially attenuated by CysA and completely prevented by CAIP (*n* = 3, Fig. 4c). These data are consistent with a Cn-mediated increase of PP1 activity in low-Na solution to dephosphorylate pS365-Cx43 and hence increase phosphorylation of S368-Cx43.

**Fig. 5:**
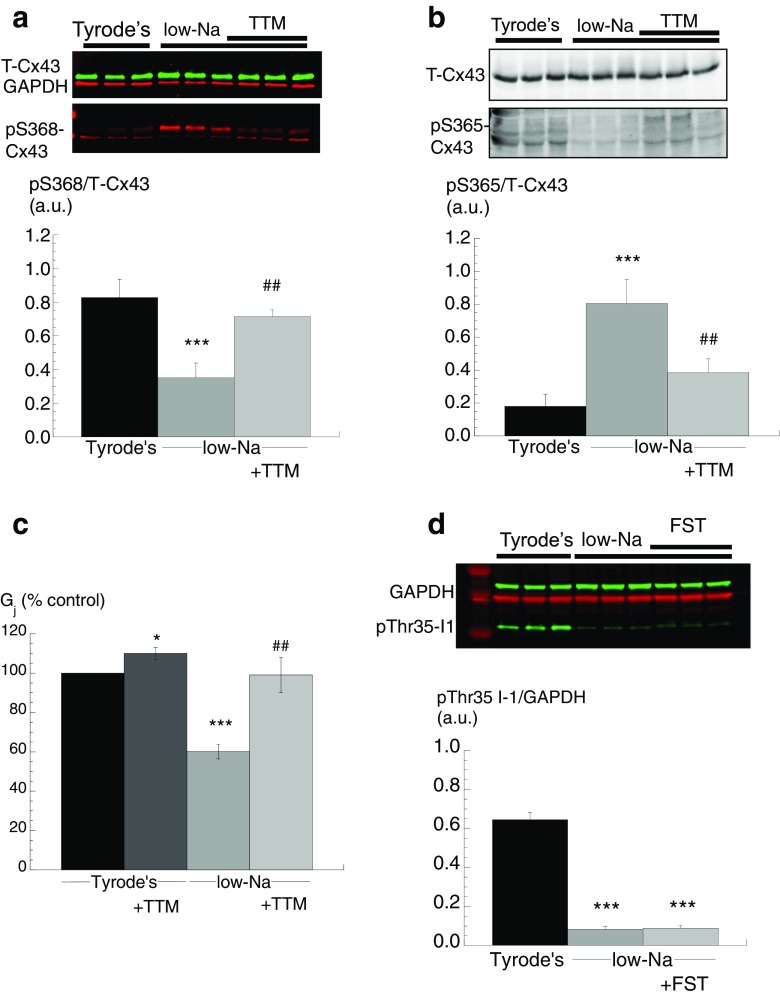
Effect of tautomycin (TTM) on Cx43 and I1 phosphorylation and on gap junction conductance, G_j_. **a** Western blots of T-Cx43, GAPDH and pS368-Cx43 in control, low-Na and low-Na with TTM. Band densities normalised to total Cx43 (T-Cx43) levels in the *lower panel*. **b** Western blots of T-Cx43 and pS365-Cx43 in control Tyrode’s, low-Na and low-Na with TTM. Band densities normalised to total Cx43 (T-Cx43) levels in the *lower panel*. **c** Values of *G*_j_ in Tyrode’s and low-Na solutions with and without TTM normalised to Tyrode’s (control). **d** Western blots of T-Cx43, GAPDH and pThr35-I1 in control Tyrode’s, low-Na, low-Na with FST. Band densities of pThr35-I1 normalised to GAPDH in lower panel. *p < 0.05 vs Tyrode’s; ***p < 0.001 vs Tyrode’s; ##*p* < 0.01 vs low-Na (*n = 3*)

Tautomycin (TTM) at a low concentration (5 nM) inhibits PP1 activity [13] and thus should prevent changes to Cx43 phosphorylation status in low-Na solution and consequent effects on *G*_j_. In low-Na solution, TTM prevented phosphorylation of S368-Cx43 (*n* = 3, Fig. 5a) and dephosphorylation of S365-Cx43 (*n* = 3, Fig. 5b). TTM had no significant effect on *G*_j_ in Tyrode’s solution; however, the decrease of *G*_j_ in low-Na solution was prevented by TTM (*n* = 6, Fig. 5c). This suggests a role for PP1 to decrease *G*_j_ when [Ca^2+^]_i_ is raised, through modulating Cx43 phosphorylation at S365.

It is important to consider also a role for PP2A, another Ca^2+^-independent protein phosphatase, that itself may influence the I1 pathway, in a way similar to that of Cn [11]. However, the PP2A-selective inhibitor fostriecin (FST, 100 nM) had no effect on pThr35-I1 levels (normalised to T-I1) in low-Na solution (*n* = 3, Fig. 5d). In addition, FST also had no effect on pThr35-I1 suggesting also that it did not affect PP2A activity. Therefore, a role for PP2A may be excluded.

### AP configuration and CV with raised [Ca^2+^]_i_—role of Cn

The above data show that a Cn-dependent pathway regulates *G*_j_ when [Ca^2+^]_i_ is raised. It has been shown previously when using thin trabeculae, as used for *G*_j_ measurement, and bipolar stimulation at one end that there is one-dimensional (1-D) AP conduction along the longitudinal axis [18]. Under these specific conditions, AP conduction is described accurately by 1-D cable theory where *G*_j_ is proportional to the square root on CV [7, 16]. This gave the opportunity to test if predictable changes to CV occurred under conditions when *G*_j_ was altered, as shown above, and investigate the role of calcineurin in any changes.

**Fig. 6:**
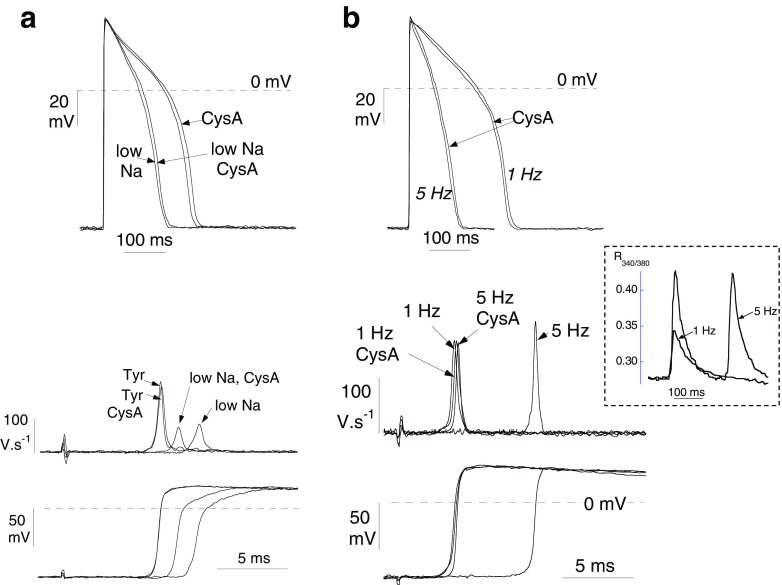
Effect of low-Na solution and increased stimulation rate on action potential (AP) morphology and conduction: effect of cyclosporin-A (CysA, 5 μM). **a** *Upper panel*: conducted APs in Tyrode’s and low-Na solution, effect of CsA. *Lower panel*: upstroke phases of the AP and (above) differential of these phases in Tyrode’s and low-Na solution, effect of CsA. **b** *Upper panel*: conducted APs in Tyrode’s at 1 and 5 Hz stimulation, effect of CsA. *Lower panel*: upstroke phases of the AP and (*above*) differential of these phases at 1 and 5 Hz stimulation, effect of CysA. *Inset* shows Fura-2 Ca^2+^ transients at 1 and 5 Hz

AP duration was slightly but significantly increased by the Cn-inhibitor CysA in all conditions (Table 1). The latency between stimulus artefact and arrival of the AP was increased in low-Na Tyrode’s solution (Fig. 6a). CysA partially reversed this increase although it had no effect in control. Measurement of latency itself cannot be used to calculate CV because the conduction pathway is uncertain near the stimulation site—and also where the preparation is attached to the force transducer. Therefore, multiple simultaneous microelectrode impalements were made at distances greater than 1 mm from the stimulation site and more than 1 mm from the attached end. CV was calculated from the ratio ∆*d*/∆*t*, where ∆*t* is the difference in latencies between separate microelectrode impalements ∆*d* apart—see “Materials and methods” section. Low-Na solution reduced CV and this was partially reversed by CysA. Values of AP duration, CV as well as d*V*/d*t*_max_ and *τ*_ap_ are listed in Table 1—d*V*/d*t*_max_ was reduced in low-Na solution but further reduced when CsA was added; *τ*_ap_ was increased in low-Na solution but unaffected by CysA. Thus, under conditions where *G*_j_ was reduced (low-Na Tyrode’s), CV was also reduced. Moreover, CysA was able to partially reverse both these reductions.

**Table 1:** Conducted AP variables in low-Na solution or at increased rate: influence of CysA

Intervention		APD, ms	d*V*/d*t*_max_, V/s	*τ*_ap_, ms	CV, cm/s	Calculated *G*_j_, mS/cm
Low-Na solution, 1 Hz stimulation	Control	218 ± 9 (9)	214 ± 15 (9)	0.24 ± 0.04 (9)	74.3 ± 6.2 (6)	4.41
	Control + CsA	228 ± 14 (9)^#^	213 ± 15 (9)	0.24 ± 0.04 (9)	72.9 ± 7.1 (6)	4.13
	Low-Na	127 ± 8 (9)*	87 ± 10 (9)*	0.53 ± 0.10 (9)*	41.8 ± 3.1 (6)*	2.52
	Low-Na + CsA	133 ± 5 (9)*^#^	71 ± 4 (9)*^#^	0.49 ± 0.09 (9)*^#^	55.3 ± 3.2 (6)*^#^	5.53
Altered stimulation rate	1 Hz	219 ± 10 (11)	215 ± 10 (11)	0.24 ± 0.04 (11)	74.3 ± 6.2 (6)	4.41
	1 Hz + CsA	228 ± 14 (11)^#^	209 ± 13 (11)	0.24 ± 0.04 (11)	72.9 ± 7.1(6)	4.13
	2 Hz	186 ± 9 (3)*	227 ± 17 (3)*	0.25 ± 0.04 (3)	62.9 ± 5.2 (3)*	2.77
	2 Hz + CsA	193 ± 9 (3) *^#^	202 ± 8 (3)^#^	0.25 ± 0.04 (3)	73.0 ± 6.1 (3)^#^	4.45
	5 Hz	115 ± 7 (11)*	245 ± 21 (11)*	0.26 ± 0.05 (11)	47.3 ± 6.9 (6)*	1.36
	5 Hz + CsA	121 ± 4 (11)*^#^	199 ± 9 (11) ^#^	0.25 ± 0.05 (11)	73.1 ± 6.5 (6)^#^	4.47

The final column lists the calculated values of *G*_j_ from mean values of CV and *τ*_ap_ (see “Discussion” section). Data are mean ± SEM; number of preparations are in parenthesis

*APD* action potential duration, *dV/dt*_max_ maximum upstroke rate of the AP, *τ*_ap_ time constant of the AP foot, *CV* conduction velocity

*p < 0.05 intervention vs control at 1 Hz stimulation

^#^CysA vs same intervention

A disadvantage of the above experiment with low-Na solution is that CV will be slowed not only by a reduction of *G*_j_. but also by attenuated inward currents in the AP upstroke which would limit the magnitude of local circuit currents. This could explain why recovery of CV with CysA was only partial. Alternatively, [Ca^2+^]_i_ was raised by increasing the stimulation rate. Increasing the rate from 1 to 2 or 5 Hz also decreased CV (and latency; Fig. 6b for 5 Hz example), and in this instance, these changes were completely reversed by CysA (Table 1). The inset shows that in isolated myocytes the intracellular Ca^2+^ transient was augmented at increased rates. With the increased rate AP duration was reduced. Moreover, d*V*/d*t*_max_ was increased and *τ*_ap_ slightly reduced, both reversed by CysA (Table 1).

## Discussion

### Intracellular [Ca^2+^], gap junction conductance and Cx43 phosphorylation status—the role of Cn

A low-Na solution was used to raise the intracellular [Ca^2+^] to about 400 nM and is sufficient to activate calcineurin [36]. Phosphorylation of Cx43 at S368 is PKC-dependent and associated with both decreased intercellular communication and reduced CV [19, 27, 39]. We confirmed this pathway in guinea-pig ventricular myocardium by showing that the PKC inhibitor CHE reversed the increase of Cx43-pS368 and the reduction of *G*_j_ by low-Na solution. Of interest also, under control conditions, CHE slightly increased resting *G*_j_ whereas Cn inhibitors had no effects. This implies that under resting conditions the value of *G*_j_ is modulated by PKC but not by Cn-dependent pathways. The role of Cn was investigated when [Ca^2+^]_i_ was raised as Cn inhibitors reversed the decrease of pS365, the increase of pS368 and the decrease of *G*_j_.

One explanation for the ability of a kinase and a phosphatase to exert the same effect on Cx43 phosphorylation at S368 and *G*_j_ is that they have different targets on the protein. A nearby site, S365, has been proposed as a gatekeeper for access to S368 so that dephosphorylation of pS365 is required to phosphorylate S368 [39]. This study identified Cn as the principal phosphatase which regulates this pathway.

### A direct or indirect effect of Cn on Cx43 phosphorylation and *G*_j_

**Fig. 7:**
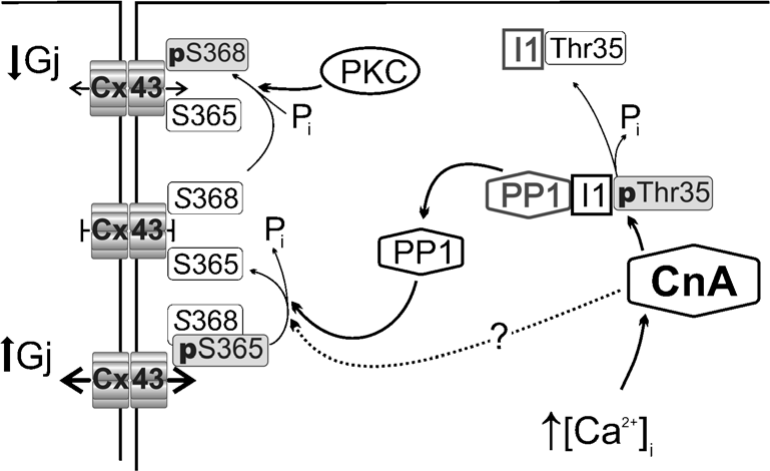
Schema of proposed Cn-dependent intracellular pathways mediating changes to Cx43 phosphorylation and gap junction conductance, G_j_. Under control conditions, Cx43 is highly phosphorylated at S365 which prevents phosphorylation of Cx43 at S368 by PKC—under this condition, *G*_j_ is high. Raised intracellular [Ca^2+^] activates Cn to dephosphorylate inhibitor 1 (I1) at Thr35 (pThr35) and the dissociation of PP1 from I1. This results in activation of PP1 to dephosphorylate pS365 enhance phosphorylation of S368 by PKC and thus decrease G_j_

The two major Ca^2+^-independent serine-threonine protein phosphatases in myocardium, PP1 and PP2A, co-localise with Cx43 at intercalated discs [1]. PP1 normally exists in an inactive complex with an inhibitor protein (I1) that in turn is phosphorylated at Thr35 (pThr35-I1) [12, 13]; dephosphorylation of I1 then releases an active form of PP1. Phosphorylated I1 (pThr35-I1) is a target for activated Cn [37] and thus potentially provides an indirect mode of action for Cn. Low-Na solution decreased pThr35-I1 levels, which in turn were reversed by CysA and CAIP. Thus, Cn-mediated dephosphorylation of S365-Cx43 could be indirectly mediated by PP1. This was corroborated by the actions of the PP1 inhibitor, TTM. It reversed the effects of low-Na solution on pS365-Cx43 dephosphorylation and S368-Cx43 phosphorylation and also predominantly reversed the reduction of *G*_j_. The pathway whereby Cn regulates Cx43 phosphorylation and the electrophysiological properties of ventricular gap junctions is summarised in Fig. 7.

TTM was used at a low concentration (5 nM) that should mainly inhibit PP1 but with potentially a smaller effect on PP2A [29]. PP2A also targets pThr35-I1 [11] and a significant rise of [Ca^2+^]_i_ may also activate this phosphatase. However, the inability of the PP2A inhibitor, fostrecin, to reverse the reduction of pThr35-I1 in low-Na solution suggests it had no role in this pathway. Moreover, there is no evidence that 5 nM TTM affects the PP2A pathway. Thus, we consider that PPI is the major downstream target activated by Cn to control gap junction phosphorylation and conductance.

### Role of protein phosphatases in modulating Cx43 phosphorylation in cardiac pathologies

The identity of the protein phosphatase(s) targeting pS365 site under pathological conditions is unclear, although some studies have proposed roles for PP1 and PP2A. Moreover, their relative importance varies with different cardiac pathologies and animal species; for example, PP1 mediates Cx43 dephosphorylation in ischaemic rat heart, but PP2A does not [22]. Alternatively, enhanced activity of PP2A, but not PP1, has been associated with human and rabbit heart failure [1]. However, in these studies, their downstream consequences on gap junction electrical properties were not measured.

Because increased Cn expression and activity occur in most cardiac pathologies, it is plausible it contributes to the final effects of PP1 and/or PP2A, as both Cn and PP2A share similar substrates, such as I1, which once dephosphorylated at Thr35 activates PP1. Moreover, increased Cx43-dephosphorylation was observed in mouse cardiomyocytes overexpressing Cn [15]. This study has clarified that in guinea-pig myocardium when [Ca^2+^]_i_ is raised, there is an interplay between Cn and PP1 to influence gap junction electrical properties and AP conduction velocity; no role for PP2A is suggested. Moreover, this study has provided new evidence for an interplay between Cn-dependent dephosphorylation of Cx43 at S365 and phosphorylation at S368 by PKC. A consequence of this is that in normal and abnormal conditions AP conduction velocity is regulated through control of gap junction conductance.

### AP conduction velocity, intracellular [Ca^2+^] and *G*_j_

Reduced CV is a crucial determinant of re-entrant arrhythmias and occurs with rapid pacing [23, 24]. With isolated preparations, as used here, AP conduction is constrained to a single dimension to allow precise delineation of the conduction pathway [7]. Moderate attenuation of CV was associated with reduction of intracellular conductance, *G*_j_. Here, it was shown that with raised [Ca^2+^]_i_, slowed conduction and reduced *G*_j_ were mediated by the Ca^2+^-CaM dependent phosphatase calcineurin. Use of the cardiac glycoside ouabain or imposition of hypoxia to presumably raise [Ca^2+^]_i_ has been shown to reduce CV as well as decrease total intracellular conductance, *G*_i_ [44, 45]. The latter is determined both by the sarcoplasmic and also gap junction conductances, and these original studies could not unequivocably attribute changes to *G*_j_, as was possible in this study. Here, two interventions were used to raise [Ca^2+^]_i_: a low-Na solution and rapid pacing, where CV and *G*_j_ could be independently measured; the former intervention was more convenient to raise [Ca^2+^]_i_ in the oil-gap chamber.

The Cn inhibitor, CysA, entirely reversed the slowed CV with rapid pacing and was partially effective in the low-Na solution. A slowed CV in low-Na solution would in part be due to reduced availability of Na^+^ current and increased dependence of inward Ca^2+^ current, so it would be expected that CysA, through an action on *G*_j_, should only partially restore CV. However, these observations are consistent with the independent demonstration that CysA, or the more specific CAIP, reversed the decrease of *G*_j_ when [Ca^2+^]_i_ was raised. Thus, these data are consistent with the hypothesis that when [Ca^2+^]_i_ is raised, Cn-dependent pathways reduce CV through a decrease of *G*_j_. It has been previously shown that rapid pacing of myocardium between 4 and 6 Hz to significantly raise [Ca^2+^]_i_ activates calcineurin [21, 43].

The actions of CysA on CV during rapid pacing and in low-Na solution are consistent with the biophysical basis of conduction [7, 8]. Rapid pacing, which reduced *G*_j_, was associated with increased d*V*/d*t*_max_ as local circuit current is concentrated nearer the propagating action potential (AP) wavefront. CysA reversed the increase of d*V*/d*t*_max_ as *G*_j_ was in turn normalised. In low-Na solution, d*V*/d*t*_max_ was decreased, due to reduced Na^*+*^ current during the AP upstroke, but was further reduced by CysA as CV itself partially recovered. This is also consistent with CysA increasing *G*_j_ under this condition.

Estimation of changes to *G*_j_ when CV when is altered in low-Na and rapid pacing conditions, in the presence and absence of CysA and under the above experimental conditions, may be made from 1-D cable theory (Eq. 1, “Materials and methods” section) and compare them when possible to actual changes of *G*_j_. CysA had no effect under control conditions but approximately halved the value in low-Na solution, as also measured in the “Results” section. CysA returned the calculated *G*_j_ to control, also consistent with the near return to normal in the “Results” section. During an increase of rate, the reduction of *G*_j_ was returned to control with CysA. Thus, the electrophysiological changes observed with increased intracellular [Ca^2+^] are consistent with calcineurin-mediated effects—reversed by CysA.

### Limitations

Measurements of AP conduction velocity and gap junction conductance, *G*_j_, of necessity used multicellular preparations. Care was taken throughout to ensure that the preparations did not develop a hypoxic core during the experiments, and a previous study found no changes to histology, ATP content or AP conduction velocity using similar preparations and over the time course of experiments carried out in this study [17]. The increase of [Ca^2+^]_i_ through rapid pacing was measured in isolated myocytes and not multicellular preparations as used to measure CV and *G*_j_; however, ion-selective electrodes do not have the temporal resolution for such measurements. CAIP was not used at as an alternative Cn inhibitor in the rapid pacing experiments where CV was slowed due to the prohibitive cost of using the agent in a rapid flow superfusion system. It is possible that in low-Na solution, there was some Ca^2+^ influx into mitochondria through its permeability transition pore (mPTP), which could lead to mitochondrial swelling and eventual cell death. However, we suggest that this is not a significant effect as all interventions using low-Na solutions had reversible effects on electrophysiological function, suggesting no damaging effects to myocytes. Although CysA blocks the mPTP, the involvement of this mechanism may not impact significantly on the results presented here.

## Electronic supplementary material

ESM 1 (DOCX 169 kb)

## Abbreviations

AP: Action potential
a.u.: Arbitrary units
CAIP: Calcineurin autoinhibitory peptide
Cn: Calcineurin
CHE: Chelerythrine
CV: Conduction velocity
CysA: Cyclosporin-A
Cx43: Connexin 43
pS368-Cx43: Phosphorylated Cx43 at serine 368
pS365-Cx43: Phosphorylated Cx43 at serine 365
d*V*/d*t*_max_: Maximum rate of action potential depolarisation
FST: Fostriecin
*G*_i_: Intracellular conductance
*G*_j_: Gap junction conductance
I1: Total protein phosphatase inhibitor-1
PKC: Protein kinase C
PP: Protein phosphatases
pThr35-I1: Phosphorylated protein phosphatase inhibitor-1 at threonine 35
S: Serine
T-Cx43: Total Cx43
TTM: Tautomycin
*z*_i_: Total longitudinal impedance
