# Supplementary material for: Regulation of gap junction conductance by calcineurin through Cx43 phosphorylation: implications for action potential conduction
Source: Pflugers Arch. 2016 Oct 19;468(11):1945–55. doi: 10.1007/s00424-016-1885-7 (PMC5138272; doi:10.1007/s00424-016-1885-7)
Supplement: Supplementary file 1 — (DOCX 169 kb) [file 424_2016_1885_MOESM1_ESM.docx]

Supplement to: Regulation of gap junction conductance by calcineurin through Cx43 phosphorylation: implications for action potential conduction.

By: Jabr RI, Hatch FS, Salvage SC, Orlowski A, Lampe PD, Fry CH

*Measurement of the intracellular [Ca^2+^], [Ca^2+^]_i_, in low-Na solution, with guinea-pig left ventricular trabeculae using intracellular Ca^2+^- and Na^+^-selective microelectrodes.*

[Ca^2+^]_i_, was recorded in isolated guinea-pig multicellular preparations, similar to those used to measure gap junction conductance and action potential conduction velocity in the present study. This was done using intracellular Ca^2+^-selective micro-electrodes (Ca^2+^-ISE) as these may be calibrated before and after use. Figure S1 shows a calibration curve of Ca^2+^-ISEs at 37°C in a mock intracellular solution containing (mM): KCl, 120.0; NaCl, 8.0; HEPES, 10.0; MgCl_2_, 1.0; EGTA, 0.5 mM, pH 7.1 with KOH.

Figure S1. Calibration curve of Ca^2+^-selective microelectrodes. Data are mean±SD from five electrodes and normalised to E_Ca_ values at 1 mM Ca in the calibrating solution. Line is a best-fit of equation 1.

The [Ca^2+^] of the calibrating solution was varied by adding samples of a 1 M CaCl_2_ stock and titrating back to pH 7.1. The [Ca^2+^] was calculated from the method in [S1]. Figure S1 shows a best-fit line through the calibration curve as described by the modified Nikolsky equation (equation 1) where *k*_ij_^pot^ is a potentiometric selectivity coefficient for Ca^2+^ (the primary ion, *i*) over the major interferent ion, Na^+^ (labelled *j*). With four electrodes *k*_ij_^pot^ had a value of 2.52±0.086.10^-3^.

*E*_Ca_ = *E*^0^ + *s*.log_10_ ([Ca^2+^]+ *k*_ij_^pot^ .[Na^+^]^2^) eqn 1

Where *E*_0_ is a constant and *s* is the slope constant of the electrode calibration, 2.303*RT/zF*, where *R*, *T*, *z* and *F* have their usual thermodynamic meanings. In equation 1, concentration values of Ca^2+^ and Na^+^ have replaced activity values, but there is no loss of generality as activity coefficients will not have varied between calibration and experimental conditions as the ionic strength of the calibration and biological media were constant throughout. A calculated ‘limit of detection’ for Ca^2+^, [Ca^2+^]_d_ - where the calibration slope is half the Nernstian slope, was calculated from equation 2 with a value of 0.25 µM when the [Na]=8 mM.

[Ca^2+^]_d_ = *k*_ij_^pot^ . [Na^+^]^2^. Eqn 2

The Ca^2+^-ISE may be used in sub-micromolar [Ca^2+^], but a separate determination of the intracellular [Na^+^] during control and low-Na conditions was necessary to calculate the steady-state intracellular [Ca^2+^] under these two conditions. From five experiments the intracellular [Na^+^] reduced from 7.2±1.2 to 2.4±0.42 mM when going from control to low-Na conditions as used in the experiments in this paper. The characteristics of Na^+^-ISEs have been described in detail previously [S2,3] and the methodology of calibration in [S1]. Therefore the steady-state concentrations of intracellular [Ca^2+^] in normal and low-Na solutions were estimated from *E*_Ca_ readings and equation S1 using the measured [Na^+^] in the two conditions.

Figure S2 shows simultaneous outputs from a 3M-KCl microelectrode, *E*_m_, and the Ca^2+^-ISE, *E*_Ca_, in a low-Na solution. On going from normal to low-Na solutions there was a small hyperpolarisation and an increase of [Ca^2+^]_i_ that was well-sustained for the duration of the intervention. The low-Na intervention was sustained for 20-30 minutes, a similar duration to that in the experiments used to measure gap junction conductance, *G*_j_, and action potential conduction velocity.

Figure S2. Measurement of membrane potential, *E*_m_, (upper trace) and the output of a Ca^2+^-sensitive microelectrode, *E*_Ca_ (lower trace, corrected for *E*_m_) ina guinea-pig left ventricular trabeculum. Where indicated the superfusate was changed from Tyrode’s solution to low-Na (29.4 mM) solution. Experiment at 37°C. The estimated [Ca^2+^]_i_ from the calibration curve, corrected for the mean change of intracellular [Na^+^], was from 71 to 358 nM.

It should be noted that on penetration of a cell with a Ca^2+^-ISE the value of the electrode output (termed *E’*_Ca_) is a measure of both *E*_m_ and the ∆[Ca^2+^]ISE, and the separate measurement of *E*_m_ with the 3M-KCl microelectrode was subtracted from *E’*_Ca_ to yield values of *E*_Ca_. Details of checking that the values of *E*_m_ recorded by the two microelectrodes are similar is given in S2. From four preparations the value of [Ca^2+^]_i_ increased from 85±10 nM to 405±105 nM.

It is important to note that with the guinea-pig preparations the increase of [Ca^2+^]_i_ was well-sustained during the low-Na intervention. This is in contrast to observations made in ferret left ventricular papillary muscles and trabeculae when only a limited transient increase of was recorded [Ca^2+^]_i_ [S2]. The latter observation agrees with transient estimates of the [Ca^2+^]_i_ in low-Na solution, made with micro-injection of aequorin into ferret right ventricular papillary muscles [S4,S5]. This indicates the suitability of guinea-pig preparations for the experiments carried out in the main study here and reflects variability of the handling of intracellular Ca^2+^ in different species. A further observation of the experiment in Figure S2 is the transient further increase of [Ca^2+^]_i_ on return to normal-Na Tyrode’s solution. This may reflect release of Ca^2+^ from mitochondria when the [Na^+^]_i_ was to normal restored via Na/Ca exchange in this organelle [S6].

References

S1 Fry CH, Langley SE. Ion-selective electrodes for biological systems. Harwood Academic Publishers, 2001. ISBN 9058231070

S2 Fry CH, Harding DP, Mounsey JP. The effects of cyanide on intracellular ionic exchange in ferret and rat ventricular myocardium. Proc R Soc, ser B 1987; 230: 53-75.

S3 Gray RP, McIntyre H, Sheridan DS, Fry CH. Intracellular sodium and contractile function in hypertrophied human and guinea-pig myocardium. Pflugers Arch 2001; 442: 117-23.

S4 Allen DG, Eisner DA, Lab MJ, Orchard CH. The effects of low sodium solutions on intracellular calcium concentration and tension in ferret ventricular muscle. J Physiol 1983; 345: 391-407.

S5 Allen DG, Eisner DA, Orchard CH. Factors influencing free intracellular calcium concentration in quiescent ferret ventricular muscle. J Physiol 1984; 350: 615-630.

S6 Fry CH, Powell T, Twist VW, Ward JP. The effects of sodium, hydrogen and magnesium ions on mitochondrial calcium sequestration in adult rat ventricular myocytes. Proc R Soc, ser B 1984: 223: 223-238.
